# *Bidens pilosa* and its active compound inhibit adipogenesis and lipid accumulation via down-modulation of the C/EBP and PPARγ pathways

**DOI:** 10.1038/srep24285

**Published:** 2016-04-11

**Authors:** Yu-Chuan Liang, Meng-Ting Yang, Chuan-Ju Lin, Cicero Lee-Tian Chang, Wen-Chin Yang

**Affiliations:** 1 Agricultural Biotechnology Research Center, Academia Sinica, Taiwan; 2 Graduate Institute of Biotechnology, National Chung-Hsing University, Taichung, Taiwan; 3 Molecular and Biological Agricultural Sciences, Taiwan International Graduate Program, Academia Sinica, Taipei, Taiwan, and National Chung-Hsing University, Taichung, Taiwan; 4 Department of Veterinary Medicine, National Chung-Hsing University, Taiwan; 5 Department of Aquaculture, National Taiwan Ocean University, Taiwan; 6 Institute of Biotechnology, National Taiwan University, Taiwan

## Abstract

Obesity and its complications are a major global health problem. In this study, we investigated the anti-obesity effect and mechanism of an edible plant, *Bidens pilosa*, and its active constituent. We first assessed the long-term effect of *B. pilosa* on body composition, body weight, blood parameters in ICR mice. We observed that it significantly decreased fat content and increased protein content in ICR mice. Next, we verified the anti-obesity effect of *B. pilosa* in ob/ob mice. It effectively and dose-dependently reduced fat content, adipocyte size and/or body weight in mice. Moreover, mechanistic studies showed that *B. pilosa* inhibited the expression of peroxisome proliferator activated receptor γ (PPARγ), CCAAT/enhancer binding proteins (C/EBPs) and Egr2 in adipose tissue. Finally, we examined the effect of 2-*β*-_D_-glucopyranosyloxy-1-hydroxytrideca-5,7,9,11-tetrayne (GHT) on adipogenesis in adipocytes. We found that *B. pilosa* significantly decreased the adipogenesis and lipid accumulation. This decrease was associated with the down-regulation of expression of Egr2, C/EBPs, PPARγ, adipocyte Protein 2 (aP2) and adiponectin. In summary, this work demonstrated that *B. pilosa* and GHT suppressed adipogenesis and lipid content in adipocytes and/or animals via the down-regulation of the Egr2, C/EBPs and PPARγ pathways, suggesting a novel application of *B. pilosa* and GHT against obesity.

Obesity is now recognized as an urgent pandemic worldwide. It was estimated that 1.9 billion people worldwide were overweighted and more than 600 million people were obese in 2014^1^. Intake of excess energy drives adipocyte hyperplasia and hypertrophy, leading to obesity^2^. Chronic obesity-related complications such as diabetes, cardiovascular disease, immune disorders and cancer impose an economic burden on patients and nations^3^,^4^.

Adipose tissues play a central role in lipid homeostasis and energy balance. Adipogenesis is a biological process characterized by morphological, cellular, and biochemical changes in adipose tissues^5^. Fibroblast-like pre-adipocytes undergo growth arrest, clonal expansion, differentiation and maturation into adipocytes by a complicated gene regulation in lipid homeostasis^5^. Multiple transcription factors such as C/EBPs and PPARγ are known to be master genes that modulate the different stages of adipogenesis in adipocytes^6^. Egr2, an upstream regulator of C/EBPs, is also implicated in adipogenesis^5^. Moreover, aP2 and adiponectin were reported to be differentiation markers in adipocytes downstream of PPARγ and/or C/EBPs and to participate in lipid metabolism and other functions although their roles need to be further investigated^7^,^8^.

In addition to diet control and physical exercise, a pharmaceutical approach is commonly used to combat obesity^9^. Current drugs used in weight loss act on reduction of fat absorption, suppression of appetite and increase of fullness^10^. Despite their efficacy, weight-loss drugs are often accompanied by undesirable side effects as well as cost-effectiveness concerns^11^. A wealth of information indicates that plants and their compounds can decrease food intake and fat absorption, increase lipid metabolism and stimulate energy expenditure^12^. Therefore, plants and their compounds are considered to be a natural, alternative way to control obesity.

*B. pilosa* is an Asteraceae plant. The Food and Agriculture Organization of the United Nations promoted its cultivation in the 1990 s because it is easy to grow, palatable and edible^13^. This plant is commonly used as a potherb or herbal medicine globally^14^. Currently, *B. pilosa* is reported to treat over 41 categories of diseases^15^. Over 200 phytochemicals have been identified from this plant, which may explain some of its pharmaceutical actions^15^. In addition to hypertension^16^,^17^, this plant^18^,^19^ and/or its polyynes^20^,^21^ has been demonstrated to treat diabetes. More recently, the use of *B. pilosa* as a nutraceutical was reported to be clinically effective against diabetes^22^. However, the anti-obesity mechanism of *B. pilosa* is not clear. In this study, we first investigated the effect of *B. pilosa* on food intake, fat content, body weight and/or adipocyte size in ICR and ob/ob mice. Further, we tested the effect of this plant on the expressional regulation of Egr2, C/EBPs and PPARγ in adipose tissues. Additionally, we examined the effect of *B. pilosa* on blood biochemistry. Finally, we studied the action of GHT, one active polyyne of *B. pilosa*, on adipogenesis and regulation of the gene expression of Egr2, C/EBPs and PPARγ in adipocytes.

## Results

### Long-term effect of *B. pliosa* on body weight, biochemical and hematological parameters and body composition in ICR mice

To explore the anti-obesity effects of *B. pilosa*, we first assessed its long-term effect on body weight, body composition, serum biochemistry and hematology in ICR mice. ICR male (Sup. Fig. S1a) and female (Sup. Fig. S1b) mice were randomly assigned into 4 groups with 5 mice per group. Four groups were fed with a standard diet (0% BP), and standard diet with 0.5% *B. pilosa* extract (BP), 1.5% BP and 2.5% BP for 24 weeks, respectively. No significant difference (*P* ≥ 0.05) in the body weight of ICR mice in either gender was observed before and after 24-wk treatment (Sup. Fig. S1a, S1b and Table 1). The food and water intake in control and *B. pilosa*-fed groups of male and female ICR mice were not statistically different (*P* ≥ 0.05, data not shown). Of note, body composition data showed that *B. pilosa* dependently reduced crude fat content in ICR females (Table 1). This reduction was more noticeable in males (Table 1). In addition, we also observed that *B. pilosa* dependently increased crude protein content in males to a greater extent than females (Table 1).

We also examined the effect of *B. pilosa* on biochemical and hematological parameters in the mouse blood of each group. The variation in biochemical and hematological parameters between the control and *B. pilosa*-fed groups was observed (Sup. Tables S1 and S2.). However, these parameters were indeed within normal range. The results suggest the safety of *B. pliosa*.

Collectively, these data showed that *B. pliosa* had an impact on body composition in mice.

### *B. pilosa* decreases body weight gain and fat content but increases lean tissue content in ob/ob mice

**Figure 1:**
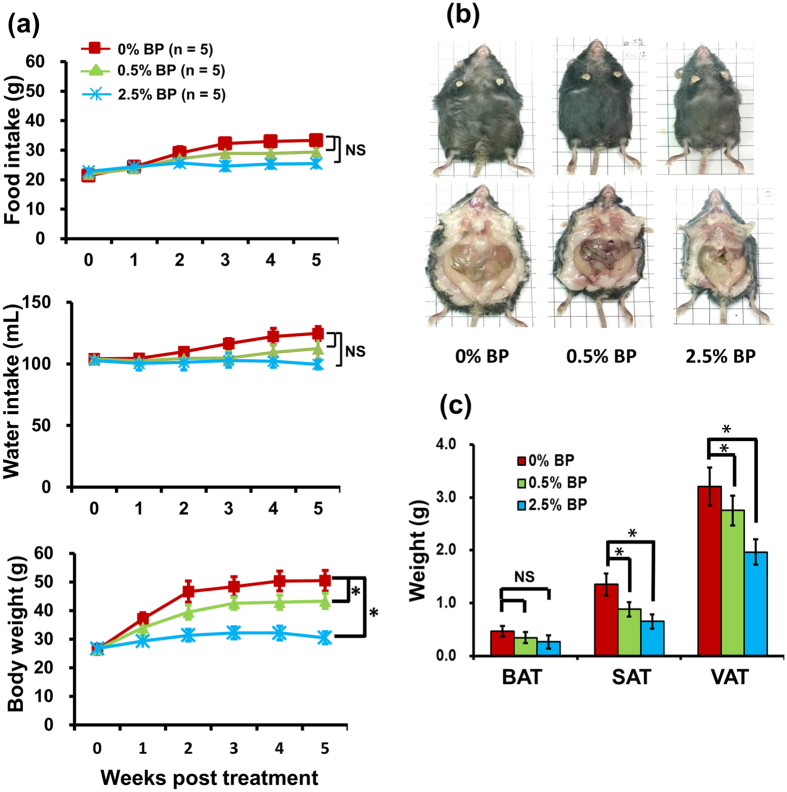
Changes in food consumption, water intake, body weight and fat tissues in male ob/ob mice fed with *B. pilosa*. (**a**) Three groups of 5-week-old mice fed with standard diet and standard diet containing 0.5% *B. pilosa* extract (BP) and 2.5% BP for 5 weeks. Food intake, water intake, and body weight were monitored weekly for 5 weeks. (**b,c**) The 10-week-old mice (**b**) and their adipose tissues (**c**) were photographed and weighed. Brown (BAT), subcutaneous (SAT) and visceral (VAT) adipose tissues are indicated. The mouse number (n) per group is indicated. ANOVA was used to compare the difference between control and treatment groups and *P* ≥ 0.05 (NS) and *P* < 0.05 (*) are shown.

**Figure 2:**
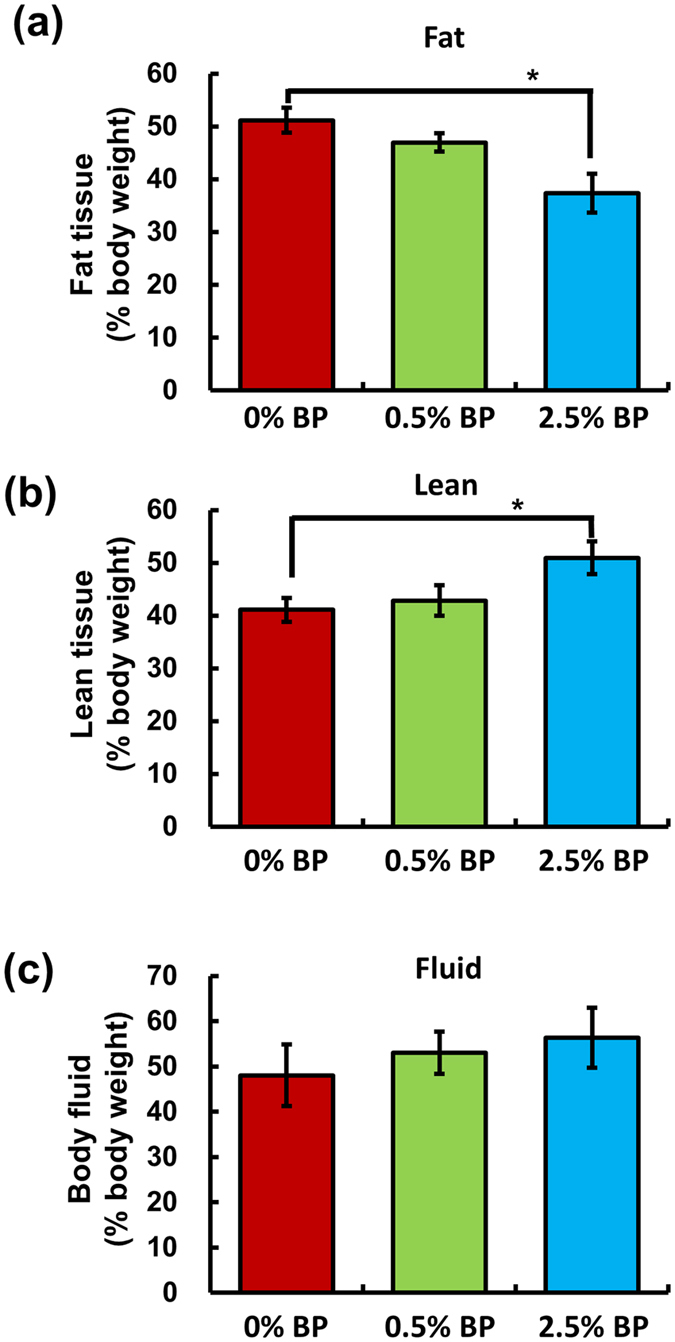
Changes in body composition of male ob/ob mice fed *B. pilosa*. Five weeks post-treatment, the mice from Fig. 1 were subjected to MRI analysis. Fat tissue, (**a**) lean tissue (**b**) and body fluid (**c**) in relation to body mass were measured and the data are presented as mean ± SEM. ANOVA was used to compare the difference between control and treatment groups and P ≥ 0.05 (NS) and *P* < 0.05 (*) are shown.

Next, the ob/ob mice, a mouse model of obesity, were used to further investigate the effect of *B. pilosa* on body weight and body composition. The 5-week-old males were randomly divided into 3 groups, 5 mice a group, fed standard diet (0% BP) and standard diet containing low (0.5% BP) or high (2.5% BP) dose of *B. pilosa* extract for 5 weeks. No significant difference (*P* ≥ 0.05) was observed in food and water consumption in the control and treatment groups of ob/ob mice (Fig. 1a). In contrast, *B. pilosa* dose-dependently decreased body weight (Fig. 1a), body fat (Fig. 1a) and serum lipids (Table 2). For adipose tissues, *B. pilosa* significantly diminished the weight of visceral and subcutaneous fat but not brown fat (Fig. 1c). Akin to the data in ICR mice, NMR data showed that *B. pilosa* decreased fat content in ob/ob mice in a dose-dependent manner (Fig. 2a). *B. pilosa* also increased the content of lean tissue in the mice (Fig. 2b). However, no difference (*P* ≥ 0.05) in the body fluid between control and *B. pilosa*-fed mice was observed (Fig. 2c).

### *B. pilosa* reduces cell size and expression level of Egr2, C/EBPs and PPARγ in adipose tissues of obese mice

**Figure 3:**
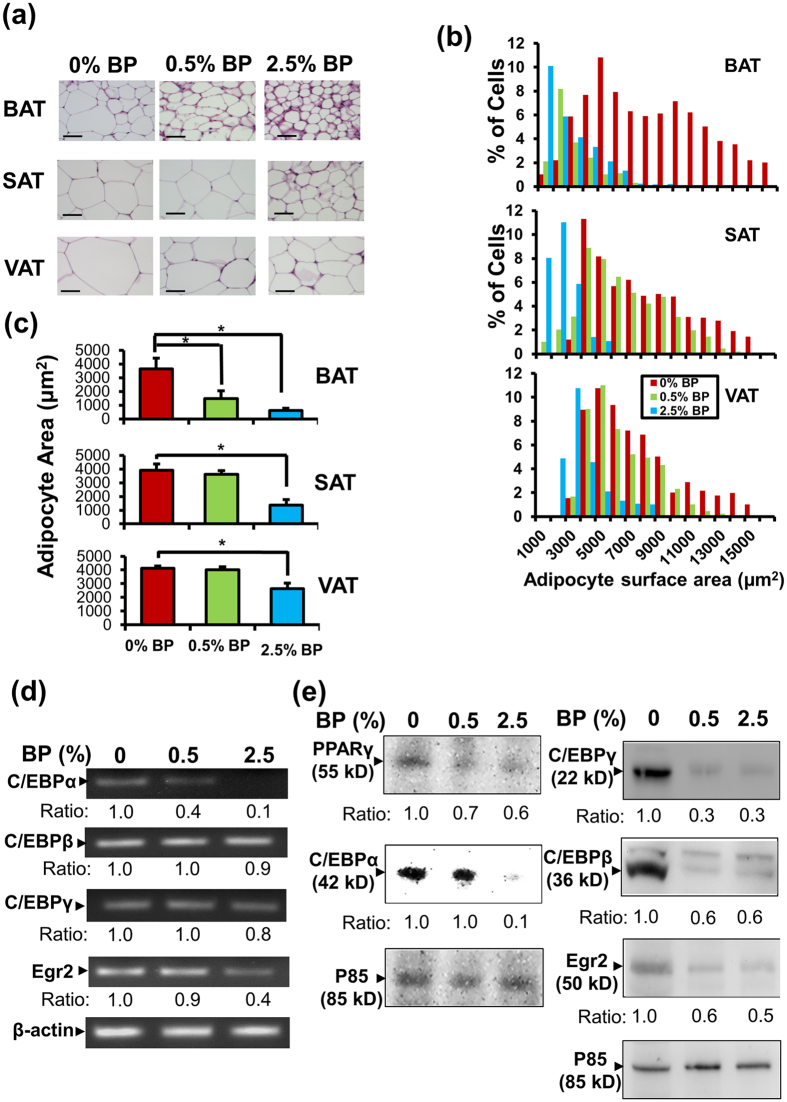
Morphological, cellular and biochemical changes in adipose tissues of male ob/ob mice fed *B. pilosa*. (**a**–**c**) Brown (BAT), subcutaneous (SAT) and visceral (VAT) adipose tissues of the ob/ob mice (Fig. 1) were collected. The adipose tissues were stained with hematoxylin and eosin (**a**). The size distribution (**b**) and average area (**c**) of adipocytes in every 100-mm^2^ area range of adipose tissues were quantified using ImageJ software. The data, expressed as mean ± SEM, was analyzed using Student’s *t*-test. P < 0.05 (*) are considered statistically significant. (**d**,**e**) Both mRNA (**d**) and protein (**e**) level of Egr2, C/EBPγ, C/EBPβ, C/EBPα and PPARγ in visceral adipose tissue (VAT) of the ob/ob mice (Fig. 1) fed with different doses of *B. pilosa* was analyzed with RT-PCR and Western blot. The ratio of each gene product to that of internal control was calculated.

To dissect the mechanism by which *B. pilosa* increased fat in mice, we first examined the effect of *B. pilosa* on adipocytes in brown, subcutaneous and visceral adipose tissue. The histochemical data revealed that *B. pilosa* dose-dependently reduced cell size of adipocytes in all three types of adipose tissue (Fig. 3a). The cell size distribution of these adipose tissues was quantified. A shift from large cell size to small cell size in adipose tissues was noted in *B. pilosa*-fed mice (Fig. 3b). Moreover, this shift was dependent on the dose of *B. pilosa* and more obvious in brown fat (Fig. 3b). Accordingly, *B. pilosa* dose-dependently diminished average adipocyte area in adipose tissues (Fig. 3c).

Since *B. pilosa* effectively reduced fat accumulation and fat cell size, we then sought to find out which of the master gene(s) involved in adipogenesis and lipid metabolism regulated by *B. pilosa* were investigated. We first explored the impact of *B. pilosa* on the transcriptional and translational levels of Egr2, C/EBPγ, C/EBPβ, C/EBPα and PPARγ in the fat tissue of control and *B. pilosa*-fed mice. The data demonstrated that *B. pilosa* suppressed the expression of Egr2, C/EBPs and PPARγ in a dose-dependent fashion (Fig. 3d,e).

### GHT inhibits adipogenesis but does not affect cell viability in (pre)adipocytes

Our previous phytochemical studies and bioassays indicated that GHT is a major compound for glycemic control in *B. pilosa* extract^20^,^21^. To further explore the molecular mechanism of *B. pilosa* in adipogenesis in this study, we, on one hand, tried to identify the likely active compound(s) in *B. pilosa* by combining phytochemisty with adipogenesis assays in mouse 3T3-L1 pre-adipocytes. Based on this bioactivity-directed fractionation and isolation strategy (Sup. Fig. S2a), we found that *B. pilosa* extract and its butanol fraction, but not water fraction, inhibited adipogenesis in mouse 3T3-L1 pre-adipocytes (Sup. Fig. S2b). We also confirmed that GHT was one active compound for suppressing adipogenesis (Sup. Fig. S2b). Besides, GHT in *B. pilosa* extract was used for quality control among the batches of *B. pilosa* (Sup. Fig. S3). On the other hand, we investigated the molecular mechanism by which *B. pilosa* inhibited adipogenesis in mouse 3T3-L1 pre-adipocytes. Consistent with the data on the adiposity in ob/ob mice (Fig. 2), *B. pilosa* decreased the transcriptional and translational levels of Egr2, C/EBPγ, C/EBPβ, C/EBPα and PPARγ (Sup. Fig. S4) in differentiating adipocytes.

**Figure 4:**
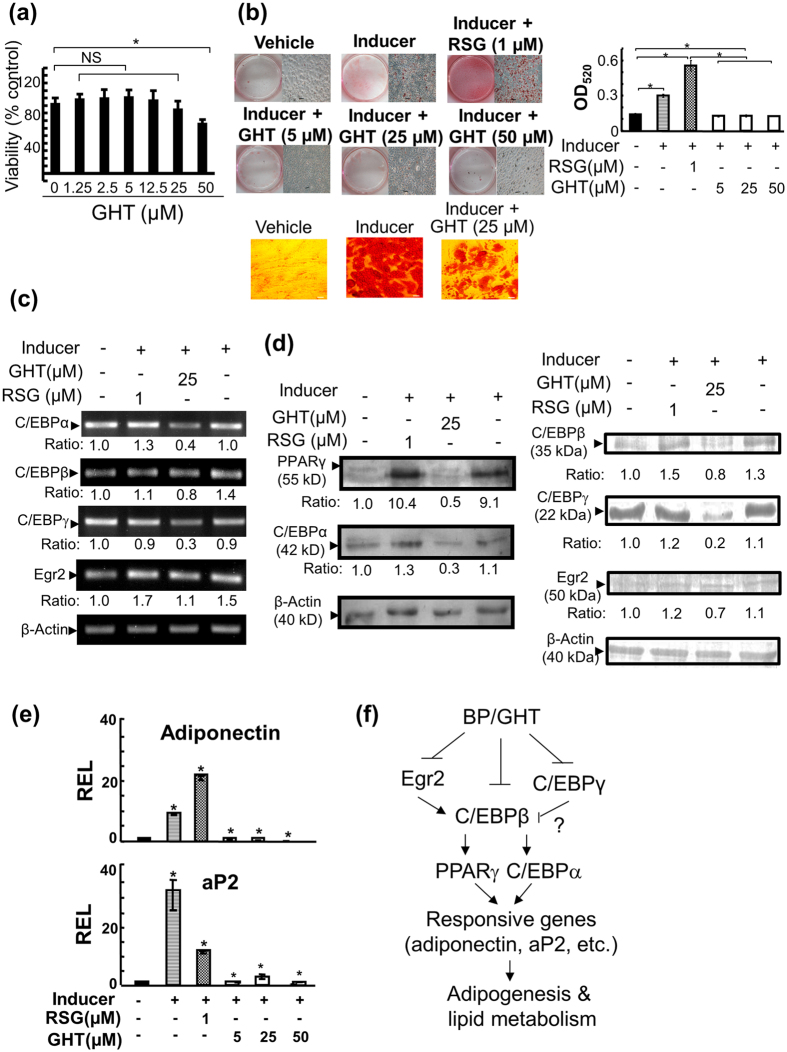
Effect of GHT, an active compound of *B. pilosa*, on differentiation of pre-adipocytes. (**a**) The MTT assay of 3T3-L1 adipocytes grown in the medium containing vehicle or GHT at the indicated concentration for 24 hr is shown. (**b**) 3T3-L1 pre-adipocytes were initiated for differentiation with DMSO (vehicle) and a mixture of Dex, IBMX and insulin for 2 days. The differentiating 3T3-L1 cells were then incubated with vehicle (inducer), rosiglitazone (RSG) or different doses of GHT until complete differentiation occurred. Lipid droplets from all the cells were visualized by Oil-Red-O staining and examined by light microscopy (left, upper panel). Scale bars = 10 μm. Oil Red O inside the cells was extracted, quantified at 520 nm and replotted into histograms (right, upper panel). Besides, human SGBS cells were incubated with DMEM/F12 medium (Vehicle), differentiation medium (Inducer) or differentiation medium in the presence of 25 μM GHT (Inducer + GHT (25 μM)) for 3 days (lower panel). Representative images of SGBS cells are shown. Scale bar = 20 μm. (**c**,**d**) Both mRNA (**c**) and protein (**d**) levels of Egr2, C/EBPγ, C/EBPβ, PPARγ and C/EBPα in 3T3-L1 cells treated with inducer in the presence of rosiglitazone (RSG) or GHT were analyzed by RT-PCR and Western blot. The ratio of the signal of each gene product to that of internal control was calculated. (**e**) The relative expression level (REL) of aP2 and adiponectin in 3T3-L1 cells was measured using real-time PCR. (**f**) A scheme delineating the possible signaling cascades of *B. pilosa* and its active compound, GHT, in adipogenesis. *B. pilosa* (BP) and GHT can reduce the expression of Egr2, an upstream regulator of C/EBPs, C/EBPγ, C/EBPβ, C/EBPα, and PPARγ, subsequently leading to the reduction of adiponectin and aP2 gene expression during adipogenesis in adipocytes. As a result, *B. pilosa* (BP) and GHT inhibit adipogenesis and lipid metabolism.

Furthermore, the effect of GHT on adipogenesis in mouse 3T3-L1 pre-adipocytes was investigated. GHT at 25 μM or less did not show cytotoxicity and a slight cytotoxicity was observed in GHT at 50 μM (Fig. 4a). Furthermore, the effect of GHT on the differentiation of 3T3-L1 pre-adipocytes into adipocytes was investigated. As expected, the inducer composed of Dex, IBMX and insulin initiated the production of lipid droplets in adipocytes compared to control cells (Vehicle vs Inducer, upper panel, Fig. 4b). Moreover, rosiglitazone enhanced this production (Inducer + RSG, upper panel, Fig. 4b). In sharp contrast, GHT dose-dependently inhibited this production (Inducer + GHT, upper panel, Fig. 4b). The inhibitory effect of GHT on the production of lipid droplets was also confirmed in human SGBS cells (lower panel, Fig. 4b).

In parallel, the effect of GHT on the transcriptional and translational levels of Egr2, C/EBPγ, C/EBPβ, C/EBPα and PPARγ in the same 3T3-L1 cells as in Fig. 4b was investigated. The 3T3-L1 pre-adipocytes had a basal expression of Egr2, C/EBPs and PPARγ at the transcriptional (Fig. 4c) and translational (Fig. 4d) levels. In contrast, the inducer significantly up-regulated the expression of these genes at the transcriptional and translational levels. Further, rosiglitazone slightly increased this up-regulation (Fig. 4c,d). However, GHT decreased the up-regulation of the expression of these genes by the inducer (Fig. 4c,d). Subsequently, the expression level of aP2 and adiponectin, two downstream genes of C/EBPs and PPARγ were examined. As expected, the inducer up-regulated the expression level of aP2 and adiponectin. In contrast, GHT reduced this up-regulation (Fig. 4e).

Overall, the data showed that *B. pilosa* and its active constituent, GHT, reduced adipogenesis in adipocytes via down-regulation of Egr2, C/EBPs and PPARγ as well as their downstream genes, aP2 and adiponectin (Fig. 4f).

## Discussion

Plants and compounds can exert anti-obesity action via reduction of appetite and fat digestion/absorption and/or increase of lipid breakdown and energy expenditure^12^. Particularly, edible plants represent an extraordinary source of foods, nutraceuticals and pharmaceuticals against obesity. In this study, we demonstrated that *B. pilosa* reduces fat content in mouse models (Sup. Fig. S1 and Fig. 2). Its anti-obesity action involves the inhibition of Egr2, C/EBPs, and PPARγ pathways in adipose tissues (Fig. 3d,e). We also studied the likely molecular basis of *B. pilosa* and GHT in differentiating adipocytes (Fig. 4, Sup. Fig. S2, and Sup. Fig. S4). Consistent with the *in vivo* data (Fig. 3), we confirmed that *B. pilosa* and GHT inhibited adipogenesis via the reduced expression of Egr2, C/EBPs, and PPARγ (Fig. 4). Although the exact molecular target of *B. pilosa* and GHT remains elusive, *B. pilosa* and GHT exerted their anti-adipogenic action via the down-regulation of the adipogenic transcriptional factors such as Egr2, C/EBPs and PPARγ as described in Fig. 4f. We also performed a bioavailability test for GHT. Following an oral administration, GHT was easily taken into the blood circulation, peaked by 30 minutes and declined within 6 hours (Sup. Fig. S5). The data suggest that GHT can be easily ingested and reach the circulation system.

*B. pilosa* is generally recognized as safe for ethnomedicinal or culinary use worldwide^13^,^15^. A dose of 400 mg *B. pilosa* extract per kilogram of body weight of three times per day, is considered safe in men^22^. Here, mice did not show any toxicity to a daily dose of *B. pilosa* extract at 27 g per kg body weight (data not shown). Consistently, this did not affect biochemical parameters and number of blood cells except for blood glucose in ICR mice (Sup. Tables S1 and S2) and blood glucose, insulin, lipids and lipoproteins in ob/ob mice (Table 2). Our data and other published literature suggest low safety risk of taking this plant orally. Aside from efficacy and palatability, *B. pilosa* is rather more cost-effective than ginseng and others due to its fast growth rate and low nutrient requirement.

High calorie diets and an inactive lifestyle drive adipocyte hyperplasia and hypertrophy, resulting in obesity. Thus, targeting adipogenesis and lipid metabolism is thought to be an anti-obesity strategy^12^,^23^. In this study, we showed that *B. pliosa* and its active compound, GHT, inhibited adipogenesis in adipocytes (Figs 3 and 4 and Sup. Fig. S4). The mechanistic studies showed that *B. pliosa* and GHT suppressed the up-regulation of gene expression of Egr2, C/EBPs, and PPARγ and their downstream genes, aP2 and adiponectin, during adipogenesis (Figs 3 and 4 and Sup. Fig. S4). Interestingly, we also observed an increase in protein content in ICR mice (Table 1) and ob/ob mice (Fig. 2b). This observation might be due to the effect of *B. pilosa* on the energy shift from fat deposition to protein deposition since food intake did not show a significant difference in control and *B. pilosa*-fed animals (Fig. 1a). It has been shown that the energy required to deposit 1 kJ of protein and 1 kJ of fat was 2.25 and 1.36 kJ in Zucker rats, respectively^24^. Therefore, unequal conversion between fat and protein in ob/ob mice by *B. pilosa* may explain the reduction in their body weight.

Overall, our data taken alone with published literature confirm the beneficial function of the edible plant, *B. pilosa*, for metabolic syndromes such as diabetes^18^,^19^,^22^, hypertension^17^ and obesity, making the use of *B. pilosa* as functional food attractive.

## Methods

### Chemicals and reagents

Dexamethasone (Dex), insulin, isobutylmethylxanthine (IBMX), Oil Red O powder, rosiglitazone (RGS), sodium bicarbonate, methylthiazoletetrazolium (MTT), Tween 20, isopropanol, formaldehyde, glutamine, glucose, sodium pyruvate, sodium bicarbonate, pantothenic acid, cortisol, triiodothyronine, methanol, and butanol were purchased from Sigma-Aldrich (St. Louis, MO, USA). Penicillin/streptomycin solution, Dulbecco’s Modified Eagle’s Medium (DMEM) and fetal bovine serum (FBS) were obtained from Hyclone Laboratories, Inc. (Logan, UT, USA) and Life Technologies Corp. (Grand Island, NY, USA). *B. pilosa* extract and GHT were produced in compliance to the good manufacturing practices guidelines and purchased from Chun-Yueh Biomedical Technology Co. (Taipei, Taiwan) as previously described^22^. Quality control of GHT in each batch of *B. pilosa* extracts was conducted as shown in Sup. Fig. S3 as previously described^22^.

### Animals and diet

Four-week-old ICR mice and male C57Bl/6J obese (ob/ob) mice were obtained from BioLASCO Taiwan (Taipei, Taiwan) and the National Laboratory Animal Center (Taipei, Taiwan), respectively. Animals were housed in a 12 h light/dark cycle in controlled temperature (22 ± 2 °C) and humidity (55 ± 10%) in a specific pathogen-free animal facility. All mice gained *ad libitum* access to rodent diet (5010, LabDiet, MO, USA) and water for 1 week prior to the study and beyond. *B. pilosa* at the indicated doses was used in the study and the number of calories in the diet supplemented with *B. pilosa* is shown in Sup. Table S3. All animal experiments were performed according to the guidelines of Academia Sinica, Taiwan. All the experimental protocols were approved by the Institutional Animal Care and Use Committee of Academia Sinica (Protocol no. 12-12-478).

### Blood biochemistry

After coagulation, mouse sera were collected from the blood samples by centrifugation at 4,000 × g for 30 min at 4 °C, and were analysed using a Fuji Dri-Chem 4000i analyzer (Tokyo, Japan). Biochemical parameters such as total cholesterol (TC), triacylglycerol (TG), uric acid (UA), high-density lipoprotein (HDL), very low-density lipoprotein (VLDL), alanine aminotransferase (ALT), aspartate aminotransferase (AST), albumin and creatinine were determined.

### Analysis of body composition

The carcasses of ICR mice were lyophilized for 48·h. The dried body mass (*M*_*b,dry*_) of each mouse was recorded and body water content was determined by subtracting *M*_b,dry_ from flushed body mass. The carcasses were ground using a Sorvall Omni-Mixer (Sorvall, Newtown, CT, USA)^25^. Fat content was determined using the ether extraction method, and protein content was measured using a Kjeltec Auto Analyzer (FOSS, Laurel, MD, USA) as published^26^. The water, fat and protein contents were expressed as a percentage of flushed body mass. To determine the body composition in live ob/ob mice, magnetic resonance imaging (MRI) system (Minispec LF50, Bruker, Ettlingen, Germany) was used to measure fat, lean tissue, and free body fluid.

### Histochemical staining

Brown, subcutaneous and visceral adipose tissues were collected from each group of ob/ob mice. All adipose samples were fixed with 10% formalin and dehydrated with a graded concentration of ethanol (70%, 80%, 90%, 95%, and 100%). After clearing in two changes of xylene, the samples were impregnated with molten paraffin wax, then embedded and blocked out. After cutting, 4- to 5-μm sections of the tissues were stained with hematoxylin and eosin and photographed under a microscope.

### Cell culture and adipogenesis

Mouse pre-adipocytes (3T3-L1 cells, CL-173) were obtained from American Type Culture Collection (ATCC, Manassas, VA, USA). Human Simpson-Golabi-Behmel syndrome (SGBS) cells were established from a patient with the Simpson-Golabi-Behmel syndrome^27^. 3T3-L1 cells were maintained in DMEM medium containing 10% FBS, 4 mM glutamine, 4.5 g/L glucose, 1 mM sodium pyruvate, and 1.5 g/L sodium bicarbonate. The differentiation of 3T3-L1 cells into adipocytes was initiated by the addition of 10 μM Dex, 0.5 mM IBMX and 10 μg/mL insulin to the confluent cells for 2 days. The cells were then differentiated in medium containing 10 μg/mL insulin together with DMSO, rosiglitazone and GHT for an additional 8 to 10 days. The medium was changed every 2 days until the differentiation was complete. For SGBS cells, the cells were cultured in differentiation medium (DMEM/F12 medium containing penicillin/streptomycin, 33 μM biotin, 17 μM pantothenic acid, 0.01 mg/mL mg transferrin, 0.1 μM cortisol, 0.2 nM triiodothyronine, 20 nM human insulin, 0.25 μM Dex, 0.5 mM IBMX and 2 μM RGS) for the first 3 days and switched to the differentiation medium lacking RGS, IBMX and Dexs for an additional 7 to 9 days^28^.

### MTT assay

MTT assay was used to determine cell viability of adipocytes. Briefly, the cells were grown in the presence of GHT at 6 × 10^3^ cells/well in a 96-well plate. After removing medium, MTT dye at 0.5 mg/mL was added to each well and incubated at 37 °C. After 4 h, DMSO (150 μL/well) was added to each well and incubated at 37 °C for additional 1 h. The plate was measured at 570 nm (signal) and 650 nm (reference) using a microplate reader (UVM340, Biochrom, UK).

### Oil Red O staining and lipid quantification

The differentiated adipocytes were extensively washed with phosphate-buffered saline (PBS) and fixed with 4% formaldehyde for 15 min. After washing with PBS, the cells were stained with 0.06% Oil Red O for 30 min. Posterior to 70% ethanol washing, the cells were photographed under a microscope. For lipid quantification, isopropanol was used to extract the intracellular oil red O, followed by measurement at 520 nm using a microplate reader.

### Western Blot

Total lysates from adipocytes or adipose tissues were electrophoresed by sodium dodecyl sulfate polyacrylamide gel electrophoresis (SDS-PAGE) and, subsequently, transferred to nitrocellulose membrane (Schleicher and Scheull, Keene, NH, USA), immunoblotted with the antibodies against PPARγ (1:2000; Santa Cruz, Dallas, TX, USA), Egr2 (1:500; Proteintech, Radnor, PA, USA), C/EBPγ (1:500; OriGene, Rockville, MD, USA), C/EBPβ (1:500; ProSci Inc., Poway, CA, USA), C/EBPα (1:1000; Abcam, Cambridge, MA, USA), p85 (1:5000; Cell Signaling Technology, Danvers, MA, USA) and/or β-actin (1:5,000; EMD Millipore, Billerica, MA, USA) and horseradish peroxidase (HRP)-conjugated goat anti-mouse IgG as secondary antibody. After developing with ECL substrate (GE Healthcare, Little Chalfont, UK), the membranes were detected using FluorChem HD2 system (Bio-Techne, Minneapolis, MN, USA).

### Reverse transcription-polymerase chain reaction (RT-PCR)

Total RNA was isolated using the Trizol Reagent (Invitrogen, Carlsbad, CA). RT-PCR was carried out with the Superscript II One-Step RT-PCR system with Taq polymerase (Invitrogen) according to the manufacture’s protocol using primer sets specific for C/EBPβ (GATGCAATCCGGATCAAACGT and AACCCCGCAGGAACATCTTT), C/EBPγ (CCCAGGGCTTGTGACTTGAA and GCCTAGAAAACAACCTCCAATGG), C/EBPα (CGTGTCCCCTCCCTTCCTGA and AGCTCCGCGGAAAAGTCTCT) and Egr2 (CGGATCTGCATGCGAAACTT and TGCGGCCACAATAGTCACAG). The PCR products were separated by agarose gel electrophoresis and visualized with ethidium bromide. Relative band intensities were quantified by ImageJ software (NIH, Bethesda, MD).

### Statistical analysis

The data are presented as mean ± standard error of the mean (SEM). Mean values were compared by analysis of variance (ANOVA) with Fisher’s least-significant difference (LSD) method for comparing groups. *P* values less than 0.05 were considered to be statistically significant.

## Additional Information

**How to cite this article**: Liang, Y.-C. *et al. Bidens pilosa* and its active compound inhibit adipogenesis and lipid accumulation via down-modulation of the C/EBP and PPARγ pathways. *Sci. Rep.* **6**, 24285; doi: 10.1038/srep24285 (2016).

## Supplementary Material

Supplementary Information

## Figures and Tables

**Table 1:** Effect of *B. pilosa* on body weight and composition of ICR mice.

**Groups**	**Initial BW (g)**	**Final BW (g)**	**Body composition**	
			**Crude protein (%)**	**Crude fat (%)**
Males				
0% BP	32.9 ± 2.1	51.6 ± 3.0	14.2 ± 1.3	19.4 ± 2.0
0.5% BP	32.3 ± 1.5	50.7 ± 3.1	15.3 ± 1.3*	17.8 ± 3.1*
1.5% BP	31.2 ± 1.5	52.1 ± 2.3	19.6 ± 1.5**	13.9 ± 2.2**
2.5% BP	32.2 ± 1.1	50.1 ± 3.6	19.3 ± 1.0**	12.8 ± 1.7**
Females				
0% BP	24.1 ± 1.4	36.1 ± 1.9	12.2 ± 1.1	22.8 ± 3.1
0.5% BP	23.8 ± 1.3	36.8 ± 2.6	13.5 ± 1.7*	20.5 ± 2.7*
1.5% BP	23.1 ± 0.9	36.5 ± 2.1	13.7 ± 1.8*	18.6 ± 2.0*
2.5% BP	23.5 ± 1.0	37.3 ± 2.8	14.3 ± 1.5**	16.5 ± 1.7**

Four groups of 5-week-old ICR males and females were fed standard diet and standard diet containing 0.5% *B. pilosa* extract (BP), 1.5% BP, and 2.5% BP for 24 wk. Body weight and composition were measured. The body composition including crude protein and fat were calculated as percentages in the dried carcass mass. The data from 5 mice per group are expressed as mean ± SEM. ANOVA was used to analyze the statistical significance. *P < 0.05 and **P < 0.01 are considered to be statistically significant when compared with control group (0% BP).

**Table 2:** Effect of *B. pilosa* on serum chemistry in ob/ob mice.

**Parameter**	**0 week**			**5 weeks**		
	**0% BP**	**0.5% BP**	**2.5% BP**	**0% BP**	**0.5% BP**	**2.5% BP**
Glucose (mg/dL)	432.4 ± 15.9	456.8 ± 19.9	430.9 ± 16.5	627.4 ± 19.8	563.4 ± 12.1*	537.3 ± 17.2*
Cholesterol (mg/dL)	149.2 ± 14.5	154.1 ± 15.3	162.3 ± 14.0	233.7 ± 15.7	161.0 ± 13.8*	144.4 ± 12.6*
Triglyceride (mg/dL)	165.3 ± 9.7	173.8 ± 8.2	171.2 ± 7.6	338.5 ± 5.9	253.6 ± 1.4*	231.6 ± 7.1*
Uric acid (mg/dL)	3.6 ± 0.3	3.1 ± 0.5	3.6 ± 0.1	4.7 ± 0.6	5.3 ± 0.1	5.1 ± 0.4
HDL (mg/dL)	123.0 ± 2.2	120.7 ± 2.4	134.3 ± 3.4	148.7 ± 2.2	92.2 ± 1.0*	83.2 ± 1.9*
VLDL(mg/dL)	38.1 ± 1.5	35.8 ± 1.7	36.2 ± 0.5	66.7 ± 1.2	50.7 ± 0.3*	44.6 ± 1.7*
Insulin (ug/dL)	0.35 ± 0.01	0.33 ± 0.01	0.34 ± 0.01	1.42 ± 0.04	0.95 ± 0.03*	0.70 ± 0.02*

Three groups of 5-week-old ob/ob mice were fed standard diet and standard diet containing 0.5% *B. pilosa* extract (BP) and 2.5% BP for 5 wk. Serum samples from 5 mice per group were collected for biochemical analysis and the data are expressed as mean ± SEM. *P < 0.05 is considered to be statistically significant when compared with control group (0% BP).
