# Supplementary material for: Bidens pilosa and its active compound inhibit adipogenesis and lipid accumulation via down-modulation of the C/EBP and PPARγ pathways
Source: Sci Rep. 2016 Apr 11;6:24285. doi: 10.1038/srep24285 (PMC4827119; doi:10.1038/srep24285)
Supplement: Supplementary Information [file srep24285-s1.doc]

***Bidens pilosa* and its active compound inhibit adipogenesis and lipid accumulation via down-modulation of the C/EBP and PPARγ pathways**

Yu-Chuan Liang^1^, Meng-Ting Yang^1,2,3^, Chuan-Ju Lin^1^, Cicero Lee-Tian Chang^4*^, Wen-Chin Yang^1,2,3,5,6*^

**Supplementary Information**

**
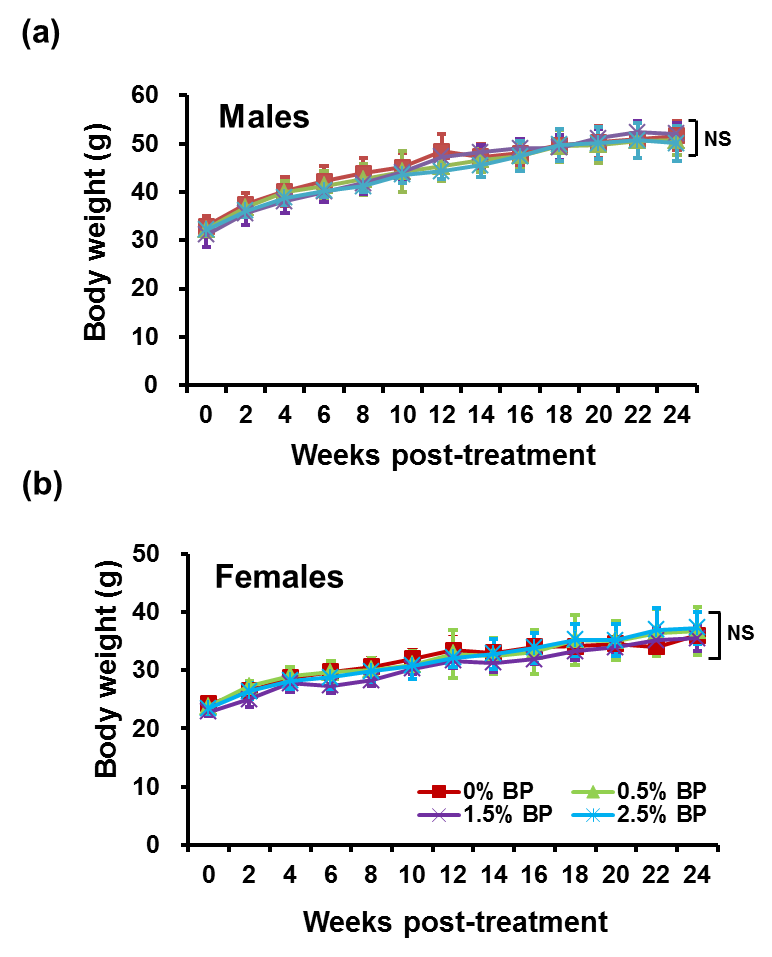
**

**Supplementary Figure S1. Changes of body weight in male (a) and female (b) ICR mice fed *B. pilosa* for 24 weeks.** All ICR mice, 5-week-old, were randomly assigned into 4 groups of males and 4 groups of females with 5 mice for each group. Each group were fed standard diet and standard diet containing 0.5% *B. pilosa* extract (BP), 1.5% BP, and 2.5% BP for 24 weeks. Body weight in each group of mice was monitored before and after 24-week treatment. The data from each group are expressed as mean ± SEM. Student’s *t*-test was used to compare the difference between control and treatment groups and *P* values of greater than 0.05 (NS) are considered to be not significant.

**
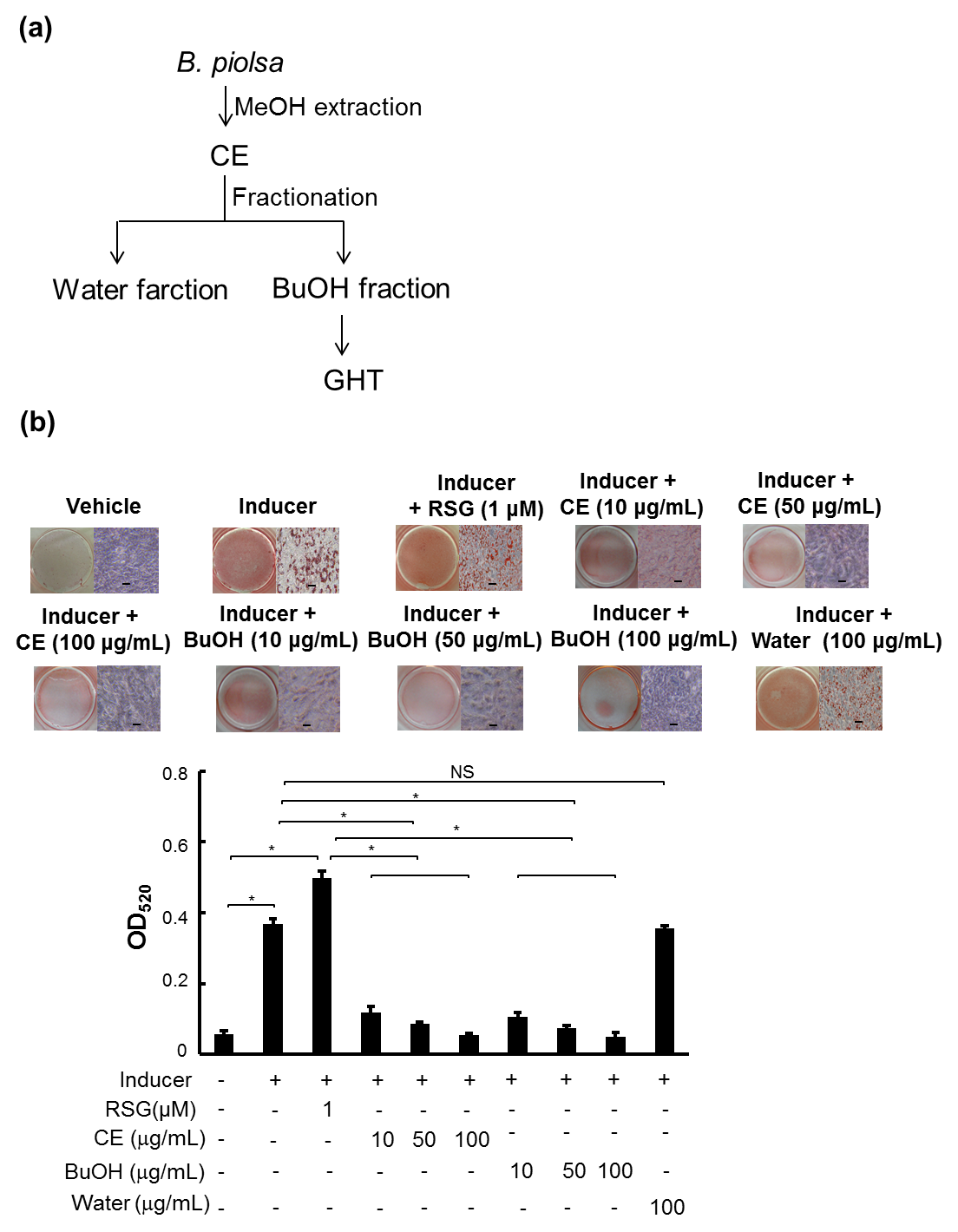
**

**Supplementary Figure S2.** **Bioactivity-directed fractionation and isolation (BDFI) of GHT.** (**a**) Flowchart of BDFI. (**b**) *B. pilosa* was extracted with methanol to yield the crude extract (CE), followed by a fractionation with water and butanol (BuOH). All the crude extract, fractions and GHT of *B. pilosa* extracts were tested for adipogenesis assays. using reverse-phase HPLC column. The peak and content of GHT were identified and quantified based on mass spectroscopy and NMR analysis [20, 21] .

**Supplementary Figure S3.** **High performance liquid chromatography (HPLC) profiles of three batches of *B. pilosa* extracts.** *B. pilosa* extracts were analyzed using reverse-phase HPLC column. The peak and content of GHT were identified and quantified based on mass spectroscopy and NMR analysis [20, 21] .


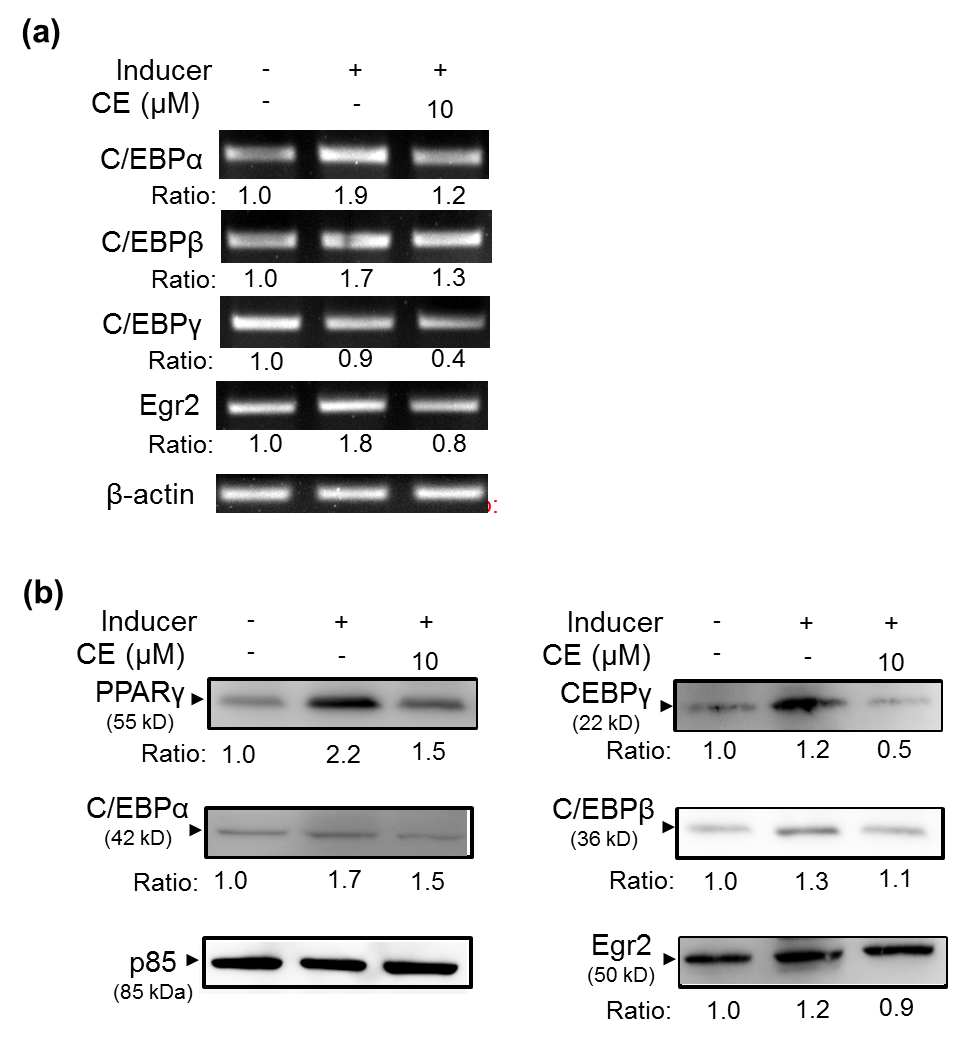


**Supplementary Figure S4. Effect of *B. pilosa* extract on differentiation of pre-adipocytes.** The same differentiating 3T3L1 cells as in the Supplementary Figure S2 were then incubated with DMEM/F12 medium (Vehicle), differentiation medium (Inducer) or differentiation medium in the presence of *B. pilosa* extract (Inducer + CE (10 μg/ml)) for 3 days until complete differentiation occurred. Both mRNA (**a**) and protein (**b**) levels of Egr2, C/EBPγ, C/EBPβ, C/EBPα and PPARγ in 3T3L1 cells were analyzed with RT-PCR and Western blot. The ratio of the signal of each gene product to that of internal control was calculated..

**
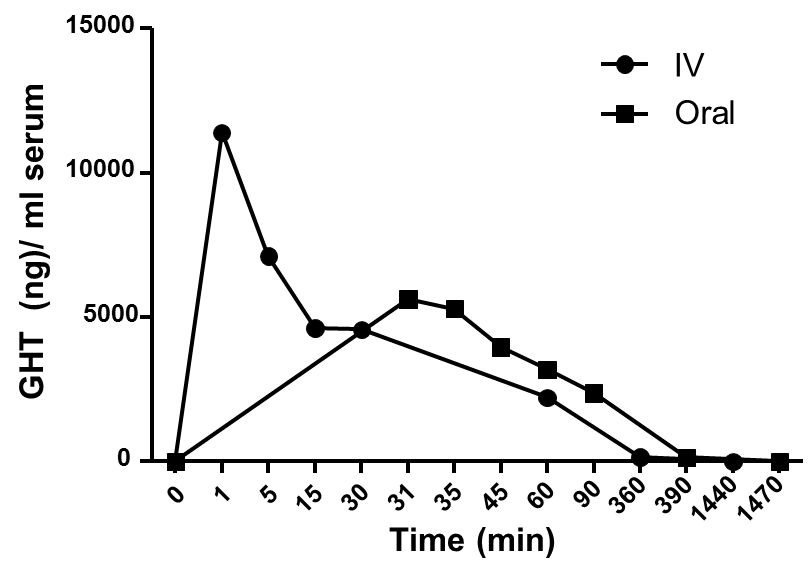
**

**Supplementary Figure S5. Blood concentration of GHT following intravenous (IV) and oral (Oral) routes of administration.** GHT was administered intravenously (1 mg/kg) and orally (25 mg/kg) to mice and GHT was measured using high pressure liquid chromatography. The average of 3 mouse samples in each time point is presented.
